# Fibrotic activity quantified in serum by measurements of type III collagen pro-peptides can be used for prognosis across different solid tumor types

**DOI:** 10.1007/s00018-022-04226-0

**Published:** 2022-03-25

**Authors:** Nicholas Willumsen, Christina Jensen, George Green, Neel I. Nissen, Jaclyn Neely, David M. Nelson, Rasmus S. Pedersen, Peder Frederiksen, Inna M. Chen, Mogens K. Boisen, Astrid Z. Johansen, Daniel H. Madsen, Inge Marie Svane, Allan Lipton, Kim Leitzel, Suhail M. Ali, Janine T. Erler, Daan P. Hurkmans, Ron H. J. Mathijssen, Joachim Aerts, Mohammed Eslam, Jacob George, Claus Christiansen, Mina J. Bissel, Morten A. Karsdal

**Affiliations:** 1 Nordic Bioscience, Herlev Hovedgade 205-207, 2730 Herlev, Denmark; 2 Bristol Myers Squibb, Princeton, NJ USA; 3 Department of Oncology, Herlev and Gentofte Hospital, Copenhagen University Hospital, Herlev, Denmark; 4 Center for Cancer Immune Therapy, Department of Oncology, Copenhagen University Hospital, Herlev, Denmark; 5 Penn State Hershey Medical Center, Hershey, PA USA; 6 Lebanon VA Medical Center, Lebanon, PA USA; 7 Biotech Research and Innovation Centre (BRIC), University of Copenhagen, Copenhagen, Denmark; 8 Department of Pathology, Erasmus University Medical Center, Rotterdam, The Netherlands; 9 Department of Medical Oncology, Erasmus MC Cancer Institute, Rotterdam, The Netherlands; 10 Department of Pulmonology, Erasmus University Medical Center, Rotterdam, The Netherlands; 11 Storr Liver Centre, Westmead Institute for Medical Research, Westmead Hospital and University of Sydney, Sydney, NSW Australia; 12 Biological Systems and Engineering Division, Lawrence Berkeley National Laboratory, Berkeley, CA 94720 USA

**Keywords:** Tumor fibrosis, Serum biomarker, Fibroblast, Collagen, Prognosis

## Abstract

Due to activation of fibroblast into cancer-associated fibroblasts, there is often an increased deposition of extracellular matrix and fibrillar collagens, e.g. type III collagen, in the tumor microenvironment (TME) that leads to tumor fibrosis (desmoplasia). Tumor fibrosis is closely associated with treatment response and poor prognosis for patients with solid tumors. To assure that the best possible treatment option is provided for patients, there is medical need for identifying patients with high (or low) fibrotic activity in the TME. Measuring unique collagen fragments such as the pro-peptides released into the bloodstream during fibrillar collagen deposition in the TME can provide a non-invasive measure of the fibrotic activity. Based on data from 8 previously published cohorts, this review provides insight into the prognostic value of quantifying tumor fibrosis by measuring the pro-peptide of type III collagen in serum of a total of 1692 patients with different solid tumor types and discusses the importance of tumor fibrosis for understanding prognosis and for potentially guiding future drug development efforts that aim at overcoming the poor outcome associated with a fibrotic TME.

## Introduction to the extracellular matrix (ECM) and collagens in the tumor microenvironment (TME)

The tumor microenvironment (TME) is important for tumor progression and patient survival. The extracellular matrix (ECM) comprises an important component of the TME in addition to the tumor cells, stromal cells and immune infiltrate [1]. The ECM is the non-cellular component of tissues and organs that provides crucial physical, bio-mechanical and bio-chemical properties that is required for tissue morphogenesis, differentiation and homeostasis [2]. The major components of the ECM are the collagens, of which 28 different types have been described, each with a unique role in supporting the tissue microarchitecture [3]. Under normal conditions, a homeostatic state of collagen turnover is maintained by a refined balance between synthesis, degradation and post-translational modifications that maintains tissue integrity. In contrast to a normal healthy stroma, this collagen homeostasis is disrupted in the TME as the composition and quality of the tumor tissue becomes altered [4]. Changes in the composition of the ECM/collagens have been shown to modulate the hallmarks of cancer and are thought to play a vital role in tumor progression and metastasis as well as in defining the likelihood of responding to anti-cancer therapies [5–8].

Overall, the ECM can be divided into the basement membrane and the interstitial matrix [2, 3]. The basement membrane underlies the epithelial and endothelial cells and supports glandular structures and blood vessels [9]. It is a relatively loose ECM with so-called network forming collagens, where type IV collagen is the most abundant protein together with laminins. The basement membrane allows nutrients and oxygen to diffuse through. In the context of cancer, loss of basement membrane structures has been associated with tumor cell invasion and angiogenesis [10, 11]. It has been well investigated and documented since the early discoveries of Mina Bissel and colleagues that the basement membrane is important for cell function and can even revert a malignant cell phenotype [12, 13]. Recent findings support that the basement membrane is key for determining the metastatic potential of cancer [14]. Cellular invasion through the basement membrane is a key factor in tumorigenesis and is driven primarily by the increased matrix metalloprotease (MMP) activity in the TME that degrade e.g. type IV collagen and alters cellular adhesion and integrin-signaling and hereby affects cell behavior [4, 15–21].

Below the basement membrane appears the interstitial matrix [2, 3]. The interstitial matrix consists of a fibrillar collagen network of type I, III, V, and XI collagens that form a 3D lattice to support tissue structure and cell function. The two major fibrillar collagens in the interstitial matrix are type I collagen and type III collagen. Type I collagen is the most abundant protein in the body and can be found in bone and connective tissues [3]. Type III collagen is the second most abundant collagen, found primarily in connective tissues.

In the TME, there is often an increased interstitial matrix deposition and remodeling of fibrillar collagens due to activation of quiescent fibroblasts into cancer-associated fibroblasts (CAFs) that not only synthesize excess amount of ECM and collagen but also contribute to MMP mediated fibrillar collagen degradation [22]. This chronic-active scarring process is also known as tumor fibrosis, or desmoplasia. As described below, tumor fibrosis has been shown to be closely associated with tumor aggressiveness, treatment response and prognosis for patients. However, we are only beginning to understand the potential impact of a fibrotic TME, the ECM and associated collagens.

## Major drivers and impact of fibrosis in the TME

The major pathological signature of tumor fibrosis is a fibrous connective tissue of interstitial matrix formed by proliferation and activation of fibroblasts which takes place inside, adjacent to, and around a solid tumor [23]. All the fibrillar collagens associated with tumor fibrosis are produced by CAFs resulting in increased deposition of a cross-linked dense and stiff collagen matrix that is impermeable for treatment, nutrients, and oxygen and therefore associated with poor outcome [24–26]. The CAF and tumor fibrosis biology builds on lessons learned from fibrotic disorders such as idiopathic pulmonary fibrosis, non-alcoholic steatohepatitis, primary sclerosing cholangitis, systemic sclerosis as well as liver, heart, lung and kidney fibrosis [4, 5, 27–35] and it has been shown that ECM turnover is generally higher in liver cancer versus cirrhosis, lung cancer versus idiopathic pulmonary fibrosis and pancreatic cancer versus chronic pancreatitis [36–38]. The CAFs promote tumorigenesis by contributing to ECM remodeling as well as secreting e.g. cytokines and growth factors to crosstalk with the immune cells and cancer cells. Among the growth factors and cytokines, transforming growth factor-β (TGF-β) is considered as the major pro-fibrotic cytokine and inducer of fibrogenesis because it promotes CAF development and increased collagen synthesis [39, 40]. Other cytokines such as interleukin (IL)-4, IL-13, and platelet-derived growth factor (PDGF) are pro-fibrotic as well and affect collagen expression [41, 42]. MMPs can also activate and release latent TGF-β stored in the ECM and hence can drive tumor fibrosis indirectly [43].

**Fig. 1:**
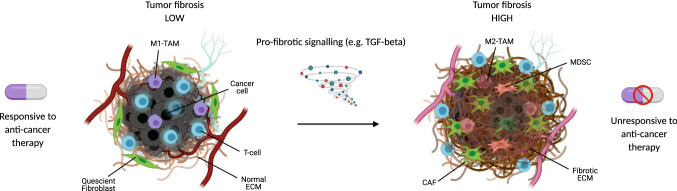
Overview of the major pathological signature of tumor fibrosis and the associated clinical impact. Tumor fibrosis is driven by pro-fibrotic signaling such as transforming growth factor beta (TGF-β) that activates quiescent fibroblasts into activated cancer associated fibroblasts (CAFs) that synthesize excess amounts of collagens resulting in a fibrotic extracellular matrix (ECM). Tumor fibrosis can be observed in many solid tumor types and forms a barrier for treatment, hindering drug penetration and T-cell recruitment to the tumor cells as well as directly impacts and regulates anti-tumor immunity due to increased recruitment of myeloid-derived suppressor cells (MDSCs) and changes in tumor associated macrophages (TAMs) composition from a pro-inflammatory (M1-TAM) to an anti-inflammatory (M2-TAM) phenotype. Fibrotic tumors are generally much less responsive to anti-cancer therapy than non-fibrotic tumors are

Tumor fibrosis may result in reduced treatment effect by forming a barrier for treatment that hinders drug penetration [26]. The interaction between tumor fibrosis, CAFs and immune cells infiltrating the tumor microenvironment directly and indirectly inhibit antitumor immunity with the activation of fibroblasts and excessive collagen deposition linked to the lack of T-cell infiltration and activity in the tumor that is a prerequisite for efficient response to immunotherapies [44–50]. This fibrosis associated T-cell exclusion from the tumor core may be due to entrapment in the collagen-rich peritumoral stroma and/or due to leukocyte-specific collagen receptor 1 (LAIR1) dependent T-cell exhaustion [48, 51, 52]. Tumor fibrosis may also limit the anti-tumor activity of effector T-cells by mediating the recruitment, and the activation of secretory programs of immunosuppressive cells such as tumor-associated macrophages (TAMs) and myeloid-derived suppressor cells (MDSCs) [47, 53, 54]. Higher collagen density within the tumor ECM promotes the polarization of TAMs to a more tumor-promoting functional phenotype characterized by enhanced expression of immunosuppressive genes and secreted proteins [55]. Furthermore, single cell sequencing studies of tumors reveal that similar to CAFs, TAMs are also capable of upregulating the expression of ECM genes, suggesting that they may themselves influence the fibrotic composition of the tumor stroma [56]. Figure 1 illustrates this major pathological tissue signature and associated clinical impact of tumor fibrosis.

## Quantifying tumor fibrosis in a liquid biopsy: potential prognostic value of measuring type III collagen pro-peptides non-invasively in patients with cancer

The common standard for assessing tumor fibrosis in patients diagnosed with cancer is by use of Sirius red or trichrome staining of total collagen content in tissue biopsies, or by staining for type I collagen and III collagen with antibodies for more detailed immunohistochemical assessments. The measurement of fibroblast activation markers, e.g. alpha smooth muscle actin (α-SMA) and fibroblast activation protein (FAP), and stromal gene signatures in the immuno-oncology setting, recently have been added to this portfolio [48, 51, 52, 56–58]

**Fig. 2:**
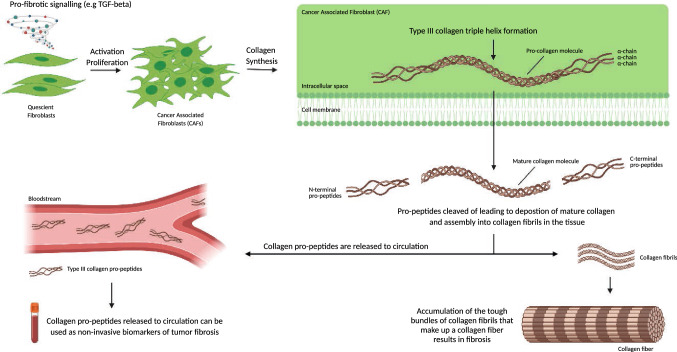
Biology and dynamics supporting the biomarker approach to quantify tumor fibrosis non-invasively. Cancer associated fibroblasts (CAFs) synthesize excess amount of fibrillar collagens such as type III collagen upon activation by for example transforming growth factor beta (TGF-β). These fibrillar collagens contain pro-peptides that are released into circulation when the collagens are deposited as collagen fibrils in the tissue. It is the excess accumulation of bundles of collagen fibrils that make up excess of collagens fibers that ultimately result in tumor fibrosis. In the blood, the pro-peptides are quantifiable biomarkers of tumor fibrosis

To describe the dynamics of tumor fibrosis, a range of novel technologies are emerging which quantify specific collagen fragments in blood [59–61]. By targeting unique fibrillar collagen degradation fragments, or pro-peptides, one may provide a dynamic measure of tumor fibrosis with the ability to quantify the collagen turnover or synthesis rate (fibrotic activity). As collagens are degraded or built into fibers, there is a release of unique epitopes that may provide information about the ongoing pathological processes of damage and repair with some epitopes being released during collagen formation (e.g. pro-peptides) and other epitopes being released during collagen degradation (e.g. MMP-generated peptide fragments) [62]. Such epitopes/peptides can be identified by mass spectrometry, then targeted by antibodies, and ultimately quantified by an immunoassay or alike. As the bone consist primarily of type I collagen, the pro-peptide from type I collagen is often used as a surrogate for bone formation whereas the degradation fragment CTX-I, is often used as a surrogate for bone degradation. As type III collagen is almost exclusively found in soft tissue and not in bone, and is derived from activated fibroblasts, it may be a superior fibrosis marker than the pro-peptide from type I collagen. An illustration of the biology and dynamics supporting this non-invasive biomarker approach to quantify tumor fibrosis is shown in Fig. 2. The rationale for investigating the prognostic value of quantifying tumor fibrosis through measurements of type III collagen fragments emerged through a hypothesis-driven approach supported by observations that type III collagen pro-peptides have been found significantly increased (> fivefold) in conditioned media from the ‘scar-in-a-jar’ in vitro culture of highly fibrotic CAFs as compared to normal fibroblast [63], and highly elevated in fibrotic disorders [4]. The monoclonal antibody used to quantify the pro-peptide of type III collagen in all the studies presented here was originally described and developed by Nielsen et al., to reflect true formation of type III collagen [64]. As this particular monoclonal antibody was raised specifically against the N-protease cleavage site of the pro-collagen (i.e. only targeting released pro-peptides) it differs from other available similar assays that either employs polyclonal antibodies or monoclonal antibodies targeting internal sequences of the pro-peptide and therefore cannot differentiate between type III collagen formation and degradation as the removal of the pro-peptide is sometimes incomplete resulting in abnormal fibrils that are prone to rapid metabolic turnover [65–68].

**Fig. 3:**
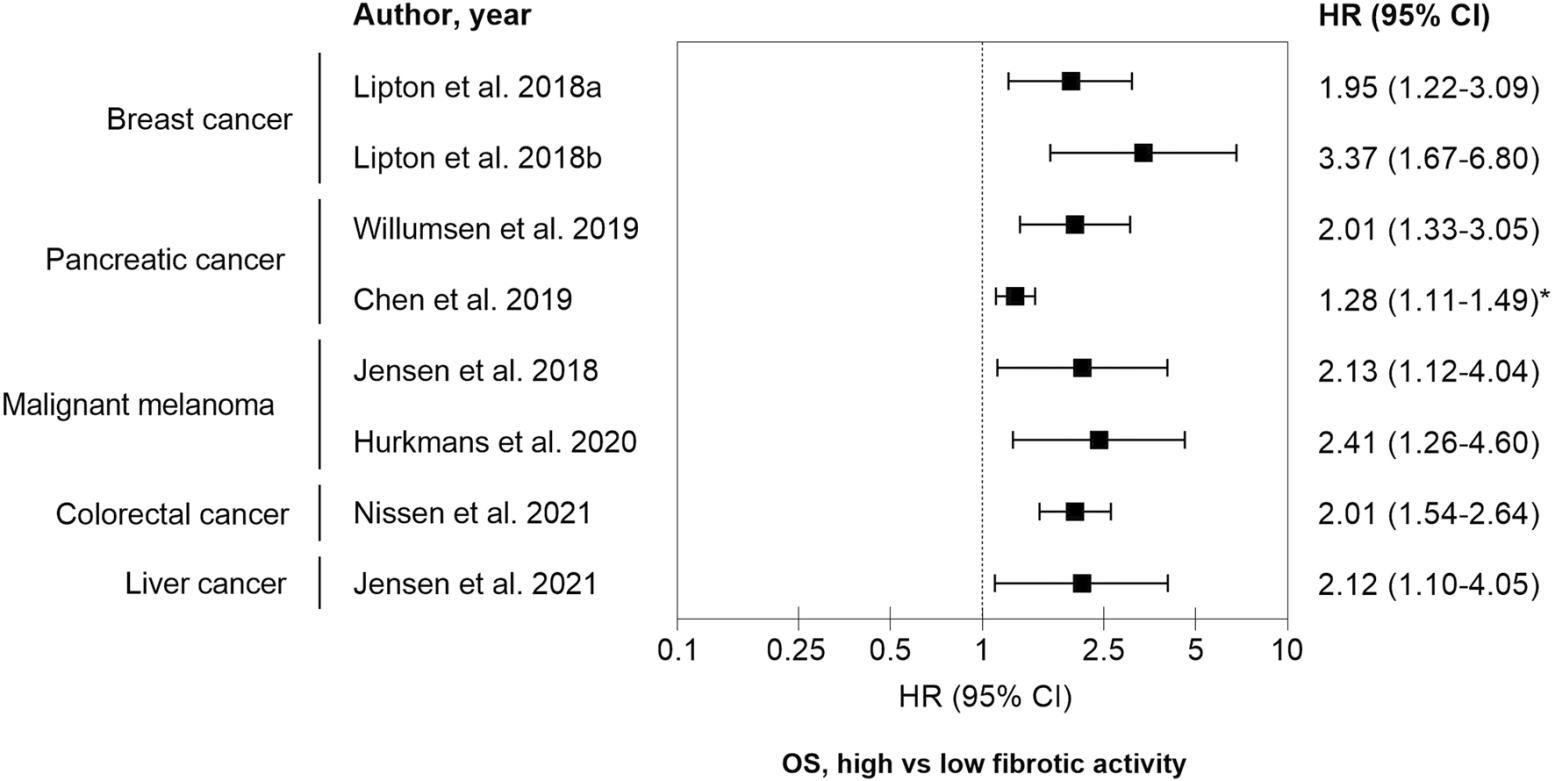
Forest plot summarizing the prognostic value of high vs low fibrotic activity. Type III collagen pro-peptides were measured in pre-treatment serum or plasma and were associated with overall survival (OS) outcomes in patients with different cancer types. All studies, except Chen et al., applied cutoffs that were based on dichotomizing patients in to ´high’ and ‘low’ levels of type III collagen pro-peptides and the exact cutoff value varied from study to study. In the study by Chen et al., the HR calculations were based on a continuous scale (*). See additional study details in Table 1

Data from clinical studies encompassing 1692 patients suffering from breast cancer, pancreatic cancer, colorectal cancer, liver cancer and malignant melanoma are summarized in the forest plot in Fig. 3. In all studies, the patients with high net fibrotic activity (type III collagen pro-peptides) at baseline had poor overall survival (OS). Approximately, two to threefold increased risk of death was observed in patients with high levels of type III collagen pro-peptides. An overview of the different patient cohorts are depicted in Table 1.

**Table 1:** Overview of clinical study cohorts evaluating pre-treatment circulating type III collagen pro-peptides as non-invasive measures of tumor fibrosis and their association with overall survival (OS)

Study	Cancer type	Therapy	No. of pts	Sample source	Cut-off	HR for OS	95% CI	*p* value
Lipton et al. [119]	Breast cancer, metastatic, ER/PR +	Letrozole	148	Serum	29.5 ng/ml	1.95	1.22–3.09	0.005
Lipton et al. [119]	Breast cancer, metastatic, HER2 +	Trastuzumab	55	Serum	25.5 ng/ml	3.37	1.67–6.80	0.001
Willumsen et al. [120]	Pancreatic cancer, advanced	Chemotherapy (5-FU)	176	Serum	10.4 ng/ml	2.01	1.33–3.05	0.001
Chen et al. [38]	Pancreatic cancer, all stages	Chemotherapy (SoC)	809	Serum	100 ng/ml increase	1.28	1.11–1.49	< 0.01
Jensen et al. [121]	Melanoma, metastatic	Ipilimumab	66	Serum	19.6 ng/ml	2.13	1.12–4.04	0.021
Hurkmans et al. [122]	Melanoma, metastatic	Nivolumab or Pembrolimumab	107	Serum	12.6 ng/ml	2.41	1.26–4.60	0.008
Jensen et al. [36]	Liver cancer, all stages	Various	79	EDTA plasma	23.9 ng/ml	2.12	1.10–4.05	0.024
Nissen et al. [123]	Colorectal cancer, metastatic	Chemotherapy + Bevacizumab	252	Serum	13.2 ng/ml	2.01	1.54–2.64	< 0.0001

*ER* estrogen receptor, *PR* progesterone receptor, *HER2* human epidermal growth factor receptor 2, *5-FU* 5-Fluorouracil, *SoC* standard of care, *HR* hazard ratio, *CI* confidence intervals

All studies, except Chen et al., applied cutoffs that were based on dichotomizing patients in to ´high’ and ‘low’ levels of type III collagen pro-peptides and the exact cutoff value varied from study to study. In the study by Chen et al., the hazard ratio (HR) calculations were based on a continuous scale and may therefore partly explain the relatively lower HR compared to the other study cohorts. Importantly, full clinical utility of type III collagen pro-peptides as a prognostic tumor fibrosis biomarker needs additional exploration of a specific cut-off per indication and treatment modality which warrants additional prospective studies. Altogether, measuring fibroblast derived type III collagen pro-peptides in serum seems not only to be a tumor agnostic, prognostic, tumor fibrosis biomarker (liquid biopsy) but also points to the need of focusing particularly on the fibroblast-derived interstitial matrix in the context of cancer [61, 63, 69, 70].

It is worth emphasizing that most of the studies listed here included patients with advanced/metastatic disease which mostly carries a very poor prognosis. Nonetheless, based on the data published from the study by Chen et al., including approximately 800 patients with pancreatic cancer this tumor fibrosis prognostic signature seems independent of stage of disease and tumor burden [38]. In addition, preliminary results of type III collagen pro-peptides measured in the early colorectal cancer setting showed associations with disease-free survival (DFS) as defined by the time interval between surgery and recurrence and is aligned with the fact that ECM composition and quality impacts and modulates the metastatic potential and hence risk of relapse (prognosis) [7]. This suggest that fibrotic activity in patients with cancer should be considered alongside more commonly assessed risk factors when attempting to provide the best possible prognosis for patients. Of interest, measuring type III collagen pro-peptides in serum was recently reported to be stable under conditions conforming with hospital sample-handling requirements and with levels not associated with sex, age, body mass index (BMI), or ethnicity [71]. In addition to the solid tumor types addressed here, elevated serum levels of collagen fragments have been found in patients with head and neck cancer, non-small cell lung cancer, gastric cancer and ovarian cancer supporting the tumor agnostic nature of altered collagen turnover and tumor fibrosis [37, 72–76].

There is a major medical need for defining this ‘fibrotic’ group of cancer patients. The first step is to differentiate those with ongoing tumor fibrosis from those without. A liquid biopsy approach as presented here for evaluating collagen peptides associated with tumor fibrosis may provide a novel and clinically applicable tool for patient stratification according to their fibrotic activity. As with any liquid biopsy, given its systemic nature there is a potential need of a concurrent (or upfront) tissue-based assessment for full histological diagnosis. However, a liquid biopsy-based approach is less invasive, quicker, and generally more frequently accessible than the gold standard tumor biopsy-based approach (which is further limited by tumor heterogeneity and is challenging, or impossible, to obtain) [77].

## Future perspectives

As highlighted above, the prognostic value of quantifying tumor fibrosis non-invasively can be obtained by measuring the pro-peptide of type III collagen in serum/plasma. The prognostic value was demonstrated across various solid tumor types including notoriously hard to treat cancers such as pancreatic cancer, and prevalent cancer types such as breast cancer, colorectal cancer, liver cancer and malignant melanoma, and for multiple treatment modalities. This supports the importance of fibrosis as a tumor agnostic process and points toward a broadly applicable biomarker approach for future clinical cancer research. While type III collagen pro-peptides is reflective of tumor fibrosis and CAF activity, type III collagen has also been shown to maintain tumor dormancy depending on context and composition [69]. Similarly, type VI collagen, another fibroblast derived collagen, can be both pro- and anti-tumorigenic, depending on context [69, 78]. In fact, there are emerging subtypes of CAFs, fibrosis types, and collagen profiles, which may have a unique function in either supporting or inhibiting cancer growth depending on context. Fibroblasts heterogeneity and the existence of different fibroblast subsets, their transcriptional profiles, and lineages are being extensively studied and where in particular iCAFs and myCAFs has been introduced as two subtypes of CAFs that play an inflammatory and myofibroblast like role, respectively, and differ in their functionality and localization within the TME [79–91]. Moreover, in several mouse models, in particular PDAC models, it has been shown that attenuating collagen synthesis in cancer associated fibroblasts increases tumor growth and spread, but at the same time may also leave tumors more prone to therapeutic intervention [92–95]. Altogether indication that there are not only good and bad fibroblast subtypes but also good and bad collagens [4].

The impact that tumor fibrosis may have on clinical outcome and in shaping the future of clinical cancer research needs to be considered. A significant percentage of patients with cancer that are included in clinical trials do not benefit from treatment, and consequently, there is a need for predictive biomarkers to treat the right patients with the right drugs at the right time [50, 96]. Intriguingly, type III collagen turnover, measured retrospectively at baseline in plasma from a discovery and validation cohort of patients with metastatic pancreatic cancer has been shown to have the capacity to also predict treatment benefit of a stromal modifier (PEGPH20) when used in combination with chemotherapy hereby providing evidence for potential predictive value [97]. In detail, both the objective response rate and survival outcomes improved significantly with PEGPH20 as an add-on to chemotherapy compared to chemotherapy alone in the patients with a high ratio of type III collagen degradation to formation, whereas in the remaining patients with a low ratio, there was no effect of adding PEGPH20 to chemotherapy. Hence, a tumor fibrosis liquid biopsy may not only be used prognostically but may also predict response to anti-fibrotic treatments. Another clinical utility may be to identify high risk patients in earlier stages of disease that may need more aggressive treatments and frequent monitoring.

In recent years, 85% of US Food and Drug Administration (FDA) approved cancer treatments have been related to the cancer-immunity cycle [98]. And while immune checkpoint inhibitors have been proven to be efficacious, underpinning the importance of the immune cells in the TME, significant differences in how inflammation is present in tumors, from complete absence to active inflammation that overlaps with the fibrotic component, have been associated with large differences in efficacy of intervention [45, 47, 56, 99–102]. Consequently, there has been much work in the immuno-oncology field to identify predictive biomarkers including measurement of programmed death-ligand 1 (PD-L1) expression, tumor mutational burden (TMB) and inflammatory gene expression profiles in the TME [99, 101–106]. While there is a common thread that such measurements are in many cases predictive of cancer immunotherapeutic efficacy, the immune responses within these microenvironments are likely to be highly complex and there is sufficient reason to believe that fibrotic activity is playing a role in establishing an immune excluded and immune suppressed TME. This may provide an opportunity for approaches to patient management that could complement current patient selection methods for improved outcomes. As measurement methods for fibrosis could potentially be deployed using conventional immunoassays and serum/plasma specimens as exemplified here, there would be significant cost and logistical advantages over current patient selection methods.

Importantly, future research is warranted to determine if altering the fibrotic status of the TME could lead to therapeutics with either monotherapy effect, enabling the host’s immune system, or in combination to enhance efficacy of anti-cancer- and immuno-therapeutics. Numerous therapeutic strategies that target aspects of tumor fibrosis to unleash the immune system against the tumor are currently under investigation [107]. For example, Jiang et al*.* have shown that modulating the fibroblast-derived collagen expression and deposition in the TME renders pancreatic cancers responsive to checkpoint inhibitor immunotherapy [108]. In other approaches, collagen-binding domains (CBDs) are being used as drug conjugates for more efficient drug delivery and reduced toxicity [109–111]. The anti-TGF-β compounds currently in clinical testing may also prove to be anti-fibrotic as part of the mode-of-action and are often tested in combination with immunotherapies [112]. As another example, losartan (an angiotensin II receptor antagonist) has been shown to inhibit type I collagen formation and reduce the desmoplastic reaction in mice with breast, pancreatic, and skin cancers and thereby enhance the efficacy of different compounds [113]. Losartan has also shown promising results in the clinic in combination with chemotherapy [114]. Likewise, metformin-induced depletion of collagen has been shown to enhance penetration of gemcitabine-loaded nanoparticles in pancreatic cancer [115]. Thus, modulating collagens and tumor fibrosis may also affect conventional treatment approaches such as chemotherapies. As the depletion of specific collagens or fibroblasts/CAFs may influence other TME components and lead to immune suppression, tumor progression and other inadvertent effects, a homeostatic restoration of the fibrotic stroma rather than its ablation may be the best approach for eliminating tumor progression [92, 95, 116–118]. Perhaps lessons learned from the organ fibrosis field can be leveraged to overcome challenges in drug development associated with tumor fibrosis.

## Conclusion

High baseline levels of type III collagen pro-peptides in serum/plasma from patients with solid tumors treated with chemotherapy, targeted therapy, or immunotherapy is a promising prognostic tumor fibrosis biomarker. These results underline the impact that tumor fibrosis may have on clinical outcome and for shaping the future of clinical cancer research towards anti-fibrotic modalities in where the type III collagen pro-peptides measured in serum/plasma could provide a new potential strategy for stratifying patients.

## Data Availability

Not applicable.

## Abbreviations

α-SMA: Alpha smooth muscle actin
BMI: Body mass index
CAF: Cancer-associated fibroblast
CBD: Collagen-binding domain
DFS: Disease free survival
ECM: Extracellular matrix
FAP: Fibroblast activation protein
FDA: Food and Drug Administration
HR: Hazard ratio
IHC: Immunohistochemistry
IL: Interleukin
LAIR1: Leukocyte-specific collagen receptor
MDSC: Myeloid-derived suppressor cell
OS: Overall survival
PDGF: Platelet derived growth factor
PD-L1: Programmed death-ligand 1
TAM: Tumor-associated macrophages
TGF-β: Transforming growth factor-β
TMB: Tumor mutational burden
TME: Tumor microenvironment
